# *HOTAIR* Induces the Downregulation of *miR-200* Family Members in Gastric Cancer Cell Lines

**DOI:** 10.52547/ibj.26.1.77

**Published:** 2021-12-20

**Authors:** Fatemeh Bossaghzadeh, Mohammadreza Hajjari, Abdolkarim Sheikhi, Iman Salahshourifar, Shiva Irani

**Affiliations:** 1 Department of Biology, Science and research Branch, Islamic Azad University, Tehran, Iran;; 2 Department of Biology, Faculty of Science, Shahid Chamran University of Ahvaz, Ahvaz, Iran;; 3 Department of Immunology, Faculty of Medicine, Dezful University of Medical Sciences, Dezful, Iran

**Keywords:** Gene expression, Long noncoding RNA, *HOTAIR*, MicroRNAs

## Abstract

**Background:**

Gastric cancer is the fourth most common human malignancy and the second reason for cancer morbidity worldwide. LncRNA *HOTAIR* has recently emerged as a promoter of metastasis in various cancer types, including GC, through the EMT process. However, the exact mechanism of *HOTAIR* in promoting EMT is unknown. Aberrant expression of the *miR-200* family has been linked to the occurrence and development of various types of malignant tumors. This study investigates the correlation between the *HOTAIR* and *miR-200* family gene expression patterns in GC cell lines. We investigated the *miR-200* and *HOTAIR* due to their common molecular features in the EMT process.

**Methods:**

AGS and MKN45 cell lines were transfected with si-*HOTAIR*, along with a negative control. The effect of *HOTAIR* knockdown was also analyzed on cell viability and also on the expression of *miR-200* family members, including *miR-200a*, -*200b*, and -*200c*, in cell lines using qRT-PCR. Statistical analysis was performed to find the potential correlation between the expression level of *HOTAIR* and miRs.

**Results:**

Our results showed significant increased *miR-200* family expression level in transfected AGS and MKN45 GC cells (fold changes > 2; *p *< 0.001). Moreover, a negative correlation was observed between *HOTAIR* and *miR-200* expression levels in GC cell lines (*p *< 0.05).

**Conclusion:**

Our findings showed a significant association between *miR-200* family and *HOTAIR* expression levels in GC cell lines. Taken together, the *HOTAIR*-*miR-200* axis seems to play a vital role in human GC, suggesting a potential therapeutic target in future GC treatment.

## INTRODUCTION

Gastric cancer is considered as one of the most common human malignancies and cancer morbidities worldwide^[^^1^^]^. Various treatment approaches such as surgery, chemotherapy and targeted therapy have continuously been developed to manage the GC patients, although the GC prognosis has still remained poor^[^^2^^-^^5^^]^. Tumor metastasis is an intricate process in which cancer cells move toward secondary organs far from the original tumor site. Metastatic cancer cells maintain their epithelial characteristics to some extents, showing mesenchymal characteristics such as invasion or distraction. This biological process is called EMT, which is characterized by the lack of E-cadherin expression, as well as N-cadherin and vimentin upregulation^[^^6^^]^.

EMT is a process that is regulated by a number of signaling pathways and transcriptional/post-transcriptional factors^[^^7^^]^, involving both miRNA and lncRNA families^[^^8^^]^. Several studies have shown the involvement of epigenetic modifications, including histone modifications, DNA methylation, lncRNAs, and miRNAs in the EMT process of cancer cells^[^^9^^-^^12^^]^. Therefore, the accumulation of genetic and epigenetic modifications can contribute to the etiology of GC^[^^13^^]^. It has been demonstrated that the major part of the human genome (98%) is non-coding DNA, which does not code for amino acids. MiRNAs and lncRNAs are two main ncRNA families and are able to control basic cellular processes through various mechanisms^[^^14^^]^. NcRNAs are classified into two major categories: structural ncRNAs and regulatory ncRNAs^[^^15^^]^. LncRNAs containing more than 200 nucleotides^[^^15^^]^, have different roles in the regulation of gene expression^[^^16^^]^. Furthermore, miRNAs are short endogenous ncRNAs of around 18–25 nucleotides length^[^^17^^,^^18^^]^ that can suppress target mRNAs involved in various biological pathways^[^^19^^]^.

Among lncRNAs, the lncRNA *HOTAIR* is a featured one recognized in 2007^[^^20^^]^. This lncRNA can repress different target genes, such as miRNAs, through an epigenetic process by recruiting polycomb repressive complex 2. Its role in different cancers, particularly gastric tumors, has been proposed previously. Silencing of *HOTAIR* has been suggested to prevent GC cell migration, invasion, and metastasis and inverses the EMT process in GC cells^[^^5^^]^. The involvement of *HOTAIR* in EMT process through epigenetically silencing of miRNAs, such as miR-34a, has previously been reported^[^^5^^]^. Nevertheless, this EMT regulatory action of *HOTAIR* on GC cells through miRNAs has not completely been understood yet.

Among miRNAs, *miR-200* family includes featured members acting as EMT regulatory factors due to their ability to cease Wnt/β-catenin pathway in GC cells^[^^21^^,^^22^^]^. The transcripts go through dynamic regulatory epigenetic changes associated with EMT or MET phenotypes during tumor progression^[^^9^^]^. It has been indicated that the *miR-200* family determines the epithelial phenotype of cancer cells by targeting the E-cadherin repressors ZEB1 and ZEB2 proteins^[^^22^^]^. By this mechanism, *miR-200* can inhibit the EMT process, which is involved in carcinogenesis. 

Based on the epigenetic regulatory effect of *HOTAIR* on different miRNAs and its potential role in EMT process and due to the regulatory effect of *miR-200* on EMT markers, our study was designed to find a correlation between the expression of *HOTAIR* and *miR-200* members. The current study was aimed to find the silencing effect of *HOTIAR* on *miR-200* members involved in EMT pathway. We found that *HOTAIR* knockdown increased the *miR-200* family level in GC cell lines. Our findings provide new insights into the molecular mechanisms of lncRNAs-mediated regulation of miRNAs involved in the EMT pathway. To our knowledge, this is the first study that reveals a correlation between the *HOTAIR* and *miR-200* family in GC. 

## MATERIALS AND METHODS

**Cell lines and culture conditions**

AGS and MKN45 human GC cell lines were prepared from the Pasteur Institute of Iran (Tehran, Iran). Cells were cultured in RPMI 1640 medium (Gibco, USA), supplemented with 10% FBS (Invitrogen, USA), and 1% penicillin and streptomycin (Invitrogen). The cells were then incubated at 37 °C, 5% CO_2_, and saturated humidity. The cells were sub-cultured when grown to suitable confluence. Cell growth was monitored using an inverted microscope. Cells in the logarithmic growth phase were cultured for further analysis.

**Knockdown of** ***HOTAIR*** **in AGS and MKN45 cell lines**

For *HOTAIR* gene silencing, AGS and MKN45 cells were transfected with 50 nM of si-*HOTAIR*; siNC was provided from Sigma (UK). The siRNAs included *HOTAIR* siRNA–SASI (Hs02_00380445) and negative control (MISSION® siRNA Universal Negative Control #1, SIGMA/SIC001). AGS and MNK45 cells were grown in 24-well plates to 50% confluence and subsequently transfected with Lipofectamine 2000 (Invitrogen) according to the manufacturer’s protocol. The experiment was repeated twice. Cells were plated until they reach 70-90% confluency at the time of transfection (AGS: 60 × 10^3^ cells and MKN45: 110 × 10^3^ cells) and then harvested at 48 h after transfection. Finally, the *HOTAIR* gene knockdown was assessed by real-time PCR. 

**Proliferation assay**

Cell viability assay was performed using MTT kit (Atocell, England), which is used to measure cellular metabolic activity as an indicator of cell viability based on the manufacturer’s guidelines. The cell lines were plated on a 96-well plate and after 24 h of seeding, AGS and MKN45 cells were transfected with 50 nM of si-*HOTAIR* along with a siNC and MOCK. MTT solution was added to each well after 48 hours and incubated in humidified atmosphere with 5% CO_2_ at 37 °C for 4 h. Colorimetric detection was carried out at 490 nm using an ELISA-reader (Bio-Rad, USA). Four replicate wells were set up in each group, and experiments were repeated two times.

**RNA extraction and cDNA synthesis from miRNAs/total RNAs**

 Total RNAs were extracted from the cultured cells using RNX (CinnaGen, Iran) based on the manufacturer’s guidelines. The extracted RNA was treated with DNaseI (Sigma) and then was stored at -80 °C. Using a Nanodrop spectrophotometer (Epoch, BioTek- USA), RNA purity was determined based on A260/280 nm absorbance ratio. In addition, RNA integrity was assessed using electrophoresis on a 1% agarose gel containing SafeStain (CinnaGen). Then RNA was reverse transcribed into cDNA using cDNA Synthesis Kit (Takara, Japan) following the manufacturer’s guidelines. The qPCR BONmiR kit (Cat no. BN-0011.17) was purchased from BonBiotech Company (Iran). The kit was applied to synthesize cDNA from all miRNAs, and SNORD gene was used as the internal control gene. Instead of using oligo dT and random hexamer, a relatively long sequence (50-70 nucleotides) called specific RTP (Provided from BonBiotech, Iran) was employed to trap miRNA and SNORD gene. In the end, the final amplification from RNAs attached to these pieces was carried out. The mixture was exposed to 16 °C for 10 minutes to bind the miRNAs to RTPs. It was then exposed to 42 °C for 40 minutes to completely amplify the components attached to the RTP. Tests were performed in duplicates.

**Real-time PCR**

Real-time PCR was performed using SYBR-Green PCR Mix (Takara) in an ABI 7100 system (Applied Biosystems, USA). The total reaction volume was 20 μl containing 1 μl of cDNA, 0.5 μl of forward primer (10 μM), 0.5 μl of reverse primer (10 μM), and 10 μl of 2× SYBR Green PCR super master mix. Conditions for PCR included denaturation at 95 °C for 5 min, 40 cycles of 5 sec at 95 °C, and annealing/extension at 60 °C for 30 s. The housekeeping gene HPRT1 was used as the internal control for *HOTAIR* gene, and SNORD gene was used as the internal control for *miR-200* family. Primers were designed by GenScript online tool (https://www.genscript.com/tools/pcr-primers-designer) and Primer-Blast (https://www.ncbi.nlm.nih. gov/tools/primer-blast/) according to the cDNA sequences extracted from the Gene bank. The primer sequences are shown in the Table 1. To validate the real-time PCR data, melt curves were plotted, and the accuracy of the curves was confirmed for each analyzed gene and primer dimer fragments. Finally, the expression of genes was assessed by 2^-^ ^Ct ^method (Livak method;  Ct: Ct gene of interest - Ct internal control) to measure the expression level of genes in each sample analyzed. The efficiency of the primers was determined through standard curves plotted by raw data.

**Statistical analysis**

Statistical analysis was performed using Graphpad Prism 8 software. Data were shown as mean ± standard deviation. Student’s t-test (unpaired) and two-way ANOVA were used for data analysis. The difference was statistically significant at *p *< 0.05. Gene expression analysis for the *HOTAIR* and *miR-200* family genes was repeated two times for each sample. 

## RESULTS

**Downregulation of** ***HOTAIR*** **expression levels in AGS and MKN45 GC by si-** ***HOTAIR***

**Fig. 1:**
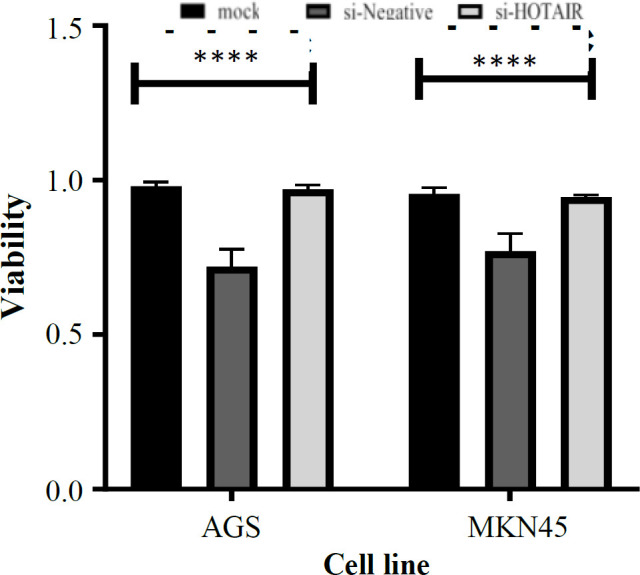
Effect of knockdown on cell viability. MTT assay was performed to evaluate the cell proliferation in si-*HOTAIR*-transfected AGS and MKN45 cell lines. The data show a statistically significant decrease in the growth of si-*HOTAIR*-transfected cells compared to siNC and MOCK groups (^****^p < 0.0001 vs. NC)

**Fig. 2:**
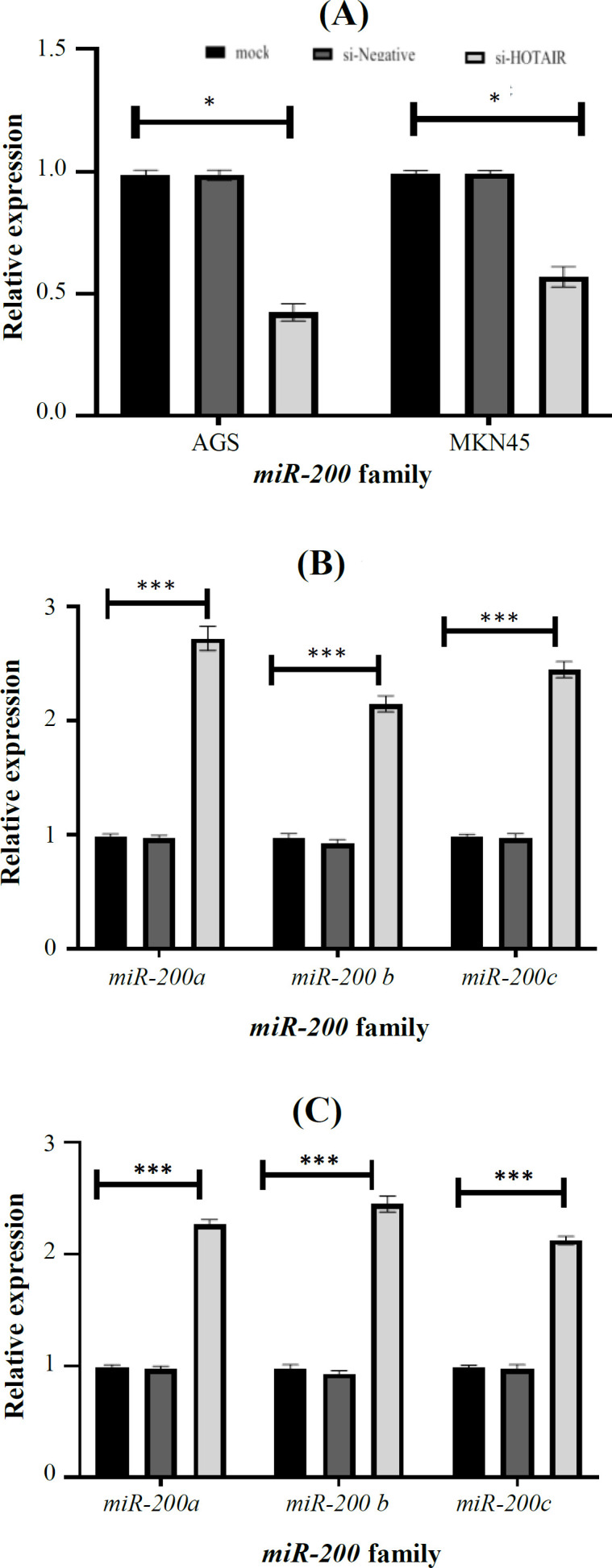
RT-qPCR indicating the downregulation of *HOTAIR* expression by si-*HOTAIR*. (A) Expression levels of *HOTAIR* in AGS and MKN45 cells transfected with si-*HOTAIR; *(B) expression levels of *miR-200c*/-*200a*/-*200b* in AGS cells transfected with si-*HOTAIR*; (C) expression levels of *miR-200a*/-200b/-200c in MKN45 cells transfected with si-*HOTAIR*. Data are expressed as mean ± SD. ^*^p < 0.05 and ^***^p < 0.001

 The knockdown of *HOTAIR* led into the decreased number of cells. There was a statistically significant reduction of si-*HOTAIR*-transfected cells growth compared to siNC and MOCK groups (for AGS = 0.28 and for MKN45 = 0.23; *p* < 0.0001; Fig. 1). After the treatment of AGS and MKN45 GC cell lines with si-*HOTAIR*, the expression level of *HOTAIR *was evaluated by qRT-PCR. As shown in Figure 2A, the expression analysis of *HOTAIR* in AGS and MKN45 transfected by si-*HOTAIR* showed the decreased expression level of *HOTAIR* in AGS and MKN45 (fold change = 0.42 and fold change = 0.57; *p* = 0.0156 and *p* < 0.05, respectively)*.*

**Table 1:** Primers used in this study

**Target gene**	**Primer name**	**Sequence**
*HOTAIR*	F R	5^’^-GAAAGGTCCTGCTCCGCTTC-3^’^ 5^’^-TCCTCTCGCCGCCGTCTG-3^’^
		
*HPRT1*	F R	5^’^-GGACTTTGCTTTCCTTGGTCAG-3^’^ 5^’^-GTCAAGGGCATATCCTACAACA-3^’^
		
*miR-200a*	F	5^’^-AACGCTAACACTGTCTGGT-3^’^
*miR-200b*	F	5^’^-TCATCCGCTAATACTGC-3^’^
*miR-200c*	F	5^’^-AGACCGCTAATACTG-3^’^
*SNORD47*	F	5^’^- ATC ACT GTA AAA CCG TT-3^’^
		
*MiR-200 *Family	Universal common reverse	[BN-0011.17.5]

*Family miR-200*, *SNORD47 *Primers Reference: Exclusively designed by Bon Yakhteh Company, Tehran, Iran; *HOTAIR, HPRT1 *Primers Reference: GenScript online tool; F, forward; R, reverse

**Altered gene expression of** ***miR-200*** **family after** ***HOTAIR*** **downregulation**

**Fig. 3:**
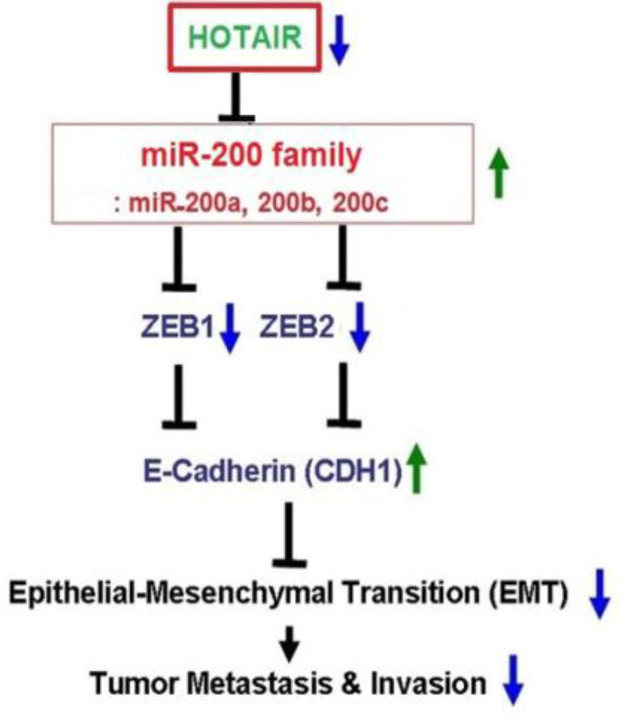
Potential mechanism involving *HOTAIR* and *miR-200* family members in the EMT process

We hypothesized that *HOTAIR* can induce EMT through the downregulation of *miR-200* family members targeting the E-cadherin repressors ZEB1 and ZEB2 proteins (Fig. 3). To examine the possible regulatory role of *HOTAIR* on *miR-200* family expression in AGS and MKN45 GC cell lines, the expression levels of three members of this family, including *miR-200a*/-*200b*/-*200c*, were analyzed. Gene expression was evaluated in AGS and MKN45 transfected with si-*HOTAIR *compared to the negative controls. RT-qPCR analysis of *miR-200* family expression levels was performed, as well. Our data revealed that the expression of *miR-200* family was upregulated in both AGS and MKN45. In other words, the knockdown of *HOTAIR* led to the increased level of all three genes of the *miR-200* family (Table 2). As shown in AGS cell line, the most differentiated expression miRNA was the *miR-200a* (fold change: 3; *p* < 0.001), while the least differentiated one was *miR-200b* gene (fold change: 2.1; *p* < 0.001), as represented in Figure 2B). Also, in MKN45 cell line, *miR-200b* gene showed the most change (fold change: 2.4; *p* < 0.001), while *miR-200c* gene reached the least change of expression (fold change: 2.1; *p* < 0.001; Fig. 2C).

**Correlations between the expression levels of** ***HOTAIR*** **and** ***miR-200*** **family**

**Fig. 4:**
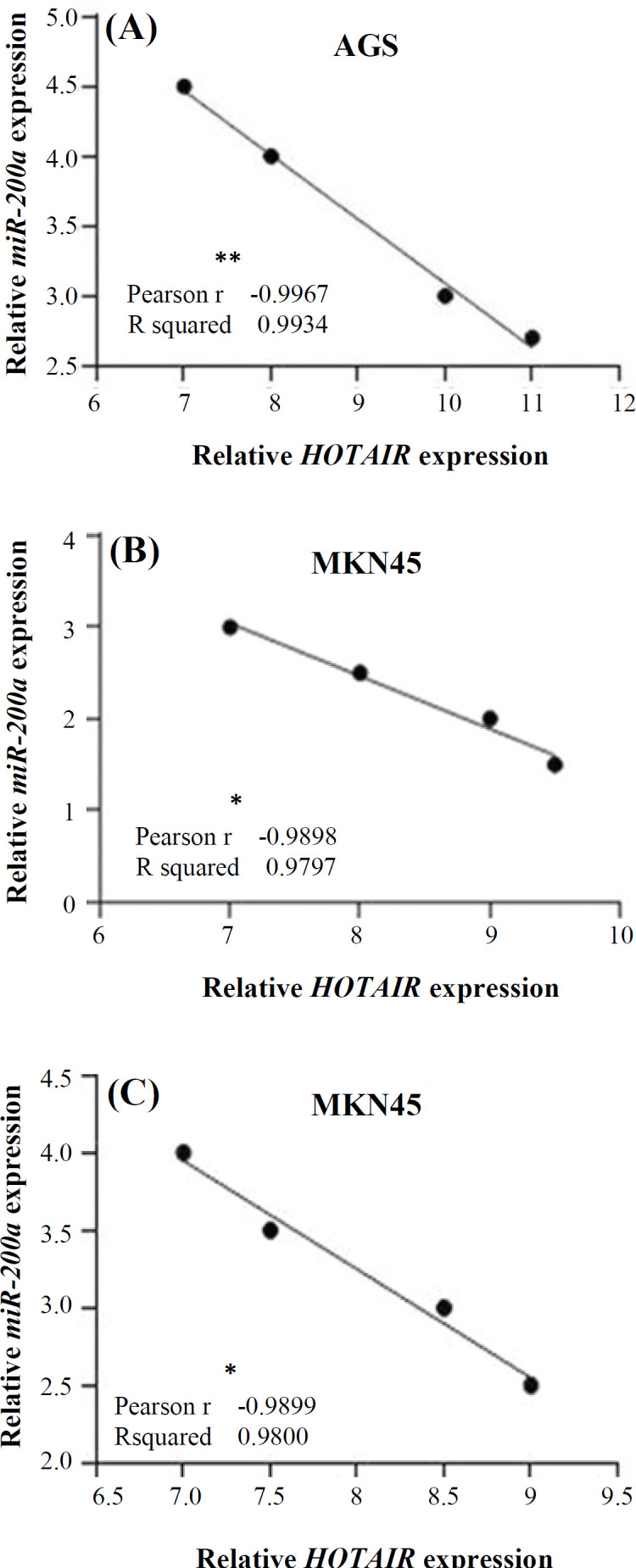
Correlation between the expression level of *HOTAIR* and *miR-200* in AGS and MKN45 GC cell lines. Pearson^’^s correlation coefficient revealed the expression levels of *HOTAIR* and (A) *miR-200a* in AGS cell line (^**^p < 0.01). (B and C) *miR-200a*/-*200c* in MKN45 cell line were negatively correlated (^*^p < 0.05)

Using Pearson's correlation coefficient, we found a correlation between the expression levels of *HOTAIR* and *miR-200* family members in GC cell lines. Statistical significant inverse correlations were observed between *HOTAIR* and *miR-200* family expression in GC cell lines. The expression levels of *HOTAIR* and *miR-200a* gene had a significant negative correlation in AGS cell line (*p* < 0.001; r = -0.9934; Fig. 4A). Furthermore, the expression level of *miR-200a*/*c* and *HOTAIR* were negatively correlated in MKN45 cell line (r = -0.9797 [Fig. 4B] and r = -0.9800 [Fig. 4C], respectively *p *< 0.05). 

## DISCUSSION

GC is known as one of the most lethal malignancies. Several studies have investigated the role of various genes in the progression of this type of cancer^[^^6^^-^^10^^,^^23^^]^. LncRNAs have recently been identified as novel regulators of transcriptional and epigenetic networks^[^^24^^]^. These RNAs achieve their biological functions through their interactions with multiple signaling molecules, including proteins^[^^25^^]^ and miRNAs^[^^26^^]^. Although being expressed in special types of cells and at specific developmental stage, lncRNAs are not often associated with different kinds of cancers^[^^27^^]^.

Since the introduction of *HOTAIR* by Rinn *et al.*^[^^20^^]^ in 2007, numerous studies have reported that the overexpression of lncRNA *HOTAIR* is associated with various cancer types^[^^28^^-^^30^^]^, suggesting the role of *HOTAIR* as an oncogene in a variety of human cancers. Previous studies have revealed that *HOTAIR* is upregulated in GC tumor tissues^[^^23^^,^^31^^-^^33^^]^. Our results confirmed that *HOTAIR* is a likely therapeutic target in the treatment of GC. However, the function of *HOTAIR* in GC has remained largely unknown. *HOTAIR* has been indicated to be able to induce EMT through repressing target genes^[^^27^^,^^34^^]^. In this study, we found the potential mechanism of action of *HOTAIR* in the downregulation of *miR-200* family members as one of the featured markers of EMT.

MicRNAs have been proved to function as important regulatory RNA molecules in tumorigenesis processes^[^^35^^]^. *MiR-200* is one of the major positive regulators in the maintenance of the epithelial phenotype via the repression of ZEB1^[^^36^^]^. The *miR-200 *family is involved in carcinogenesis through the regulation of EMT and is regulated through DNA methylation as a gene-silencing approach^[^^37^^]^. A few studies have indicated that *miR-200* family can be downregulated in epithelial cancers, particularly GC^[^^38^^-^^41^^]^. The role of *miR-200* has previously been reported as a tumor suppressor factor for the inhibition of the EMT process and tumor growth of GC through targeting ZEB1 and ZEB2^[^^22^^]^.

The role of *HOTAIR* in EMT regulation of GC has demonstrated in various investigations^[^^5^^,^^34^^,^^42^^]^. *HOTAIR* epigenetically represses numerous factors including miR-34a; and contributes to the process of GC cell EMT^[^^5^^,^^41^^] ^by recruiting the PRC2^[^^5^^]^. Previous surveys have also indicated the role of *HOTAIR*^[^^27^^,^^34^^]^ and *miR-200* family^[^^9^^,^^41^^]^ in promoting EMT program in GC. As *HOTAIR* can silence the expression of *miR-200* family, we hypothesize a correlation between *HOTAIR* and *miR-200* in GC. Our findings suggested a novel mechanism mediating shifts between EMT and MET programs through *HOTAIR* and *miR-200* members. Therefore, *miR-200 *and *HOTAIR* were investigated in this study due to their common molecular features in EMT process. 

Our results showed that *HOTAIR* knockdown increases the expression level of *miR-200a*, *miR-200b*, and *miR-200c* in both AGS and MKN45 GC cell lines. In this study, we explored the correlation between *HOTAIR* knockdown and *miR-200* family expression.

Our data signified that the level of *miR-200* family in GC cell lines is closely associated with the *HOTAIR *expression level. A negative correlation was also observed between *HOTAIR* and *miR-200* expression levels in GC cell lines. As *miR-200* family determines the epithelial phenotype of cancer cells by targeting the E-cadherin repressors ZEB1 and ZEB2 proteins^[^^9^^]^, our results displays that *HOTAIR* induces the migration and metastasis of GC cells through *miR-200* family, thereby affecting EMT. We speculate that *HOTAIR* has an effect on the EMT through this mechanism (Fig. 3). However, further studies are needed to reveal the exact regulatory mechanism of *HOTAIR* on this miR family. Overexpression of *HOTAIR* as well as interaction analyses between *HOTAIR* and regulatory factors/elements can help the researchers to find more about this axis. 

**Table 2:** Fold change and *p *value of expression analysis of *miR-200* family in the loss of function study of *HOTAIR* in different GC cell lines

***p *** **value**	***AGS***	***MKN45***	**Gene**
0.001	FG: 3	FG: 2.3	*miR-200a*
0.001	FG: 2.1	FG: 2.4	*miR-200b*
0.001	FG: 2.4	FG: 2.1	*miR-200c*

FG, fold change

Our investigation highlights the need for understanding the mechanism involving biological processes and the regulatory interactions between ncRNAs and coding transcripts, which may be beneficial to GC treatment. *HOTAIR*-*miR-200* axis seems to play an important role in human GC, reflecting a promising therapeutic target in future GC treatment. To our knowledge, this is the first study reporting the *HOTAIR*-*miR-200* axis in GC. However, the exact mechanism should be explored by more surveys in future. 

## CONFLICT OF INTEREST.

None declared. 
